# Anti-PD-1 monoclonal antibody MEDI0680 in a phase I study of patients with advanced solid malignancies

**DOI:** 10.1186/s40425-019-0665-2

**Published:** 2019-08-22

**Authors:** Aung Naing, Jeffrey Infante, Sanjay Goel, Howard Burris, Chelsea Black, Shannon Marshall, Ikbel Achour, Susannah Barbee, Rena May, Chris Morehouse, Kristen Pollizzi, Xuyang Song, Keith Steele, Nairouz Elgeioushi, Farzana Walcott, Joyson Karakunnel, Patricia LoRusso, Amy Weise, Joseph Eder, Brendan Curti, Michael Oberst

**Affiliations:** 1 Department of Investigational Cancer Therapeutics, University of Texas MD Anderson Cancer Center, Houston, TX 77030 USA; 2 Drug Development Unit, Sarah Cannon Research Institute, Tennessee Oncology, Nashville, TN USA; 3 Department of Medical Oncology, Montefiore Medical Center, Albert Einstein College of Medicine, Bronx, NY USA; 4 Translational Medicine, Oncology R&D, AstraZeneca, Gaithersburg, MD USA; 5 Department of Research, Amplimmune Inc., Gaithersburg, MD USA; 6 Discovery Sciences, Oncology R&D, AstraZeneca, Gaithersburg, MD USA; 7 Clinical Pharmacology & Safety Sciences, BioPharmaceuticals R&D, AstraZeneca, Gaithersburg, MD USA; 8 Oncology Biometrics, Oncology R&D, AstraZeneca, Gaithersburg, MD USA; 9 Early Oncology Clinical, Oncology R&D, AstraZeneca, Gaithersburg, MD USA; 10 Department of Hematology & Oncology, Karmanos Cancer Institute, Wayne State University, Detroit, MI USA; 11 Medical Oncology, Yale Cancer Center, New Haven, CT USA; 12 Earle A Chiles Research Institute, Providence Cancer Institute, Portland, OR USA; 13 Present Address: Department of Oncology, Janssen, Raritan, NJ USA; 14 Present Address: PRA Health Sciences, Blue Bell, PA USA; 15 Present Address: Early Oncology Clinical, Oncology R&D, AstraZeneca, Gaithersburg, MD USA; 16 Present Address: Department of Immuno-Oncology Research, FivePrime Therapeutics, Inc., South San Francisco, CA USA; 17 Present Address: Late-stage Development, Oncology R&D, AstraZeneca, Gaithersburg, MD USA; 18 Present Address: Department of Clinical Development, Arcus Biosciences, Hayward, CA USA; 19 Present Address: Medical Oncology, Yale Cancer Center, New Haven, CT USA

**Keywords:** MEDI0680, PD-1, Immunotherapy, Kidney cancer, Melanoma

## Abstract

**Background:**

The safety, efficacy, pharmacokinetics, and pharmacodynamics of the anti-programmed cell death-1 antibody MEDI0680 were evaluated in a phase I, multicenter, dose-escalation study in advanced solid malignancies.

**Methods:**

MEDI0680 was administered intravenously once every 2 weeks (Q2W) or once every 3 weeks at 0.1, 0.5, 2.5, 10 or 20 mg/kg. Two cohorts received 20 mg/kg once a week for 2 or 4 weeks, then 20 mg/kg Q2W. All were treated for 12 months or until progression. The primary endpoint was safety. Secondary endpoints were efficacy and pharmacokinetics. Exploratory endpoints included pharmacodynamics.

**Results:**

Fifty-eight patients were treated. Median age was 62.5 years and 81% were male. Most had kidney cancer (*n* = 36) or melanoma (*n* = 9). There were no dose-limiting toxicities. Treatment-related adverse events occurred in 83% and were grade ≥ 3 in 21%. Objective clinical responses occurred in 8/58 patients (14%): 5 with kidney cancer, including 1 with a complete response, and 3 with melanoma. The relationship between dose and serum levels was predictable and linear, with apparent receptor saturation at 10 mg/kg Q2W and all 20 mg/kg cohorts.

**Conclusions:**

MEDI0680 induced peripheral T-cell proliferation and increased plasma IFNγ and associated chemokines regardless of clinical response. CD8+ T-cell tumor infiltration and tumoral gene expression of *IFNG, CD8A*, *CXCL9*, and granzyme K (*GZMK*) were also increased following MEDI0680 administration.

**Trial registration:**

NCT02013804; date of registration December 12, 2013.

**Electronic supplementary material:**

The online version of this article (10.1186/s40425-019-0665-2) contains supplementary material, which is available to authorized users.

## Introduction

MEDI0680, previously named AMP-514, is a humanized IgG4κ anti-programmed cell death-1 (PD-1) monoclonal antibody developed to block the immune suppressive PD-1 pathway. The binding of tumoral programmed cell death ligand-1 and -2 (PD-L1 and PD-L2) to the PD-1 receptor on T cells suppresses their ability to launch an antigen-specific antitumor immune response [1–3]. PD-1 expression increases on T cells when they are activated, and increased PD-1 expression on circulating T cells has been associated with poor clinical outcome [4]. Blockade of this ligand binding permits continued activation of T cells and has been associated with clinical efficacy in cancer patients [5, 6].

In recent years, antagonistic monoclonal antibodies (mAbs) targeting PD-1 and PD-L1 have demonstrated the ability to restore T-cell effector function and reduce tumor progression [4, 5]. PD-1 targeted immunotherapies nivolumab, pembrolizumab, and cemiplimab have been approved in multiple solid tumor indications [7–9]. Among these, melanoma and kidney cancer, specifically renal cell carcinoma (RCC), are considered two of the most immunogenic types of cancer. The efficacy of PD-1-directed therapies in melanoma may be linked to the high mutational burden associated with this cancer type [10, 11]. Although kidney cancer has a lower mutational burden than melanoma [11], nivolumab has shown encouraging results in clinical trials and has been approved in the US for RCC [8].

Despite encouraging clinical activity, many patients do not respond to anti-PD-1 mAb therapy or relapse after an initial response, including some patients with evidence of pretreatment PD-L1 expression, immune-cell infiltration, or intermediate-to-high tumor mutational burden [12]. Combinations of anti-PD-1 agents with other immunotherapy agents may offer an opportunity to overcome some of these barriers to response to anti-PD-1 monotherapy. Several combinations are being investigated in ongoing clinical trials, including nivolumab with BMS-986253 (an anti-interleukin-8 mAb; NCT03400332), ALT-803 (an interleukin-15 superagonist complex; NCT02523469), and interferon-gamma (IFNγ) (NCT02614456) [13–15] and pembrolizumab with p53MVA, an antitumor vaccine (NCT03113487, NCT02432963) [16]. Another possible combination is with an anti-PD-L1 mAb. Currently, two clinical trials combining anti-PD-1 with anti-PD-L1 agents are ongoing, including one with MEDI0680 (NCT02936102 and NCT02118337) [17, 18]. The biological rationale for this combination approach is simultaneous blockade of PD-1/PD-L1/PD-L2 and PD-1/PD-L1/CD80 interactions [19–21]. The purpose of the current study is to characterize the initial safety and clinical efficacy of this anti-PD-1 mAb, and to confirm its intended pharmacodynamic activity.

Nivolumab and pembrolizumab have shown different safety and efficacy profiles in varying tumor types, despite sharing the same mechanism of action [22–27]. Antagonistic antibodies targeting the same protein may have the same mechanism of action, but differences in immunogenicity, binding affinity, plasma half-life, and tissue penetration could affect clinical efficacy, safety, and pharmacokinetics [28–30]. Anti-PD-1 mAbs vary due to the degree of antibody humanization and sequence differences in their complementarity-determining regions (CDRs), which determine the precise epitopes bound on the target [28]. MEDI0680 differs from nivolumab and pembrolizumab in its CDR sequence and affinity, which may impact its safety or clinical activity.

Here we present the clinical results of the dose-escalation phase of the first-time-in-human (FTIH) phase I study of MEDI0680, including safety, tolerability, and efficacy in patients with solid tumors (NCT02013804). We also describe the preclinical characterization of MEDI0680, as well as its pharmacokinetic and pharmacodynamic profiles in patients.

## Materials and methods

### Patients and study design

In this open-label, multicenter, dose-escalation and expansion study in checkpoint inhibitor-naïve patients with advanced solid malignancies, MEDI0680 was administered intravenously every 2 weeks (Q2W) or every 3 weeks (Q3W) at doses of 0.1, 0.5, 2.5, 10 or 20 mg/kg as indicated in Table 1. Two cohorts received 20 mg/kg every week (QW) for 2 or 4 weeks followed by 20 mg/kg Q2W. Patients were enrolled using a 3 + 3 study design. One cycle of treatment was defined as 21 days for patients on the Q3W schedule and as 28 days for patients on the Q2W schedule. Key eligibility criteria for the study are shown in Additional file 1: Table S1.

**Table 1:** Baseline patient characteristics

	Q3W (mg/kg)					Q2W (mg/kg)		QW × 2 (mg/kg)	QW × 4 (mg/kg)	Total
	0.1 (n = 5)	0.5 (*n* = 5)	2.5 (n = 3)	10 (*n* = 6)	20 (*n* = 9)	10 (*n* = 4)	20 (*n* = 18)	20 (*n* = 3)	20 (*n* = 5)	*N* = 58
Median age, years (range)	61 (23–69)	62 (54–74)	58 (51–69)	65 (55–78)	59 (46–81)	68.5 (46–75)	65.5 (48–86)	65 (61–70)	59 (50–64)	62.5 (23–86)
Male, n (%)	5 (100)	4 (80)	2 (67)	4 (67)	8 (89)	3 (75)	13 (72)	3 (100)	5 (100)	47 (81)
Race, n (%)										
White	5 (100)	5 (100)	3 (100)	6 (100)	9 (100)	4 (100)	16 (89)	3 (100)	5 (100)	56 (97)
Asian	0	0	0	0	0	0	1 (6)	0	0	1 (2)
Other	0	0	0	0	0	0	1 (6)	0	0	1 (7)
ECOG status, n (%)^a^										
0	3 (60)	4 (80)	2 (67)	2 (40)	4 (44)	2 (50)	8 (44)	2 (67)	2 (40)	29 (51)
1	2 (40)	1 (20)	1 (33)	3 (60)	5 (56)	2 (50)	10 (56)	1 (33)	3 (60)	28 (49)
Median time from primary diagnosis to study entry, months (range)	48 (4–80)	10 (8–27)	66 (34–140)	26 (9–54)	18 (5–46)	47 (5–137)	15 (0–109)	72 (40–72)	32 (8–57)	19 (0–140)
Smoking history, (%)										
Never smoked	3 (60)	1 (20)	2 (67)	3 (50)	4 (44)	2 (50)	8 (44)	0	3 (60)	26 (45)
Former/current smoker	2 (40)	4 (80)	1 (33)	3 (50)	5 (56)	2 (50)	10 (56)	3 (100)	2 (40)	32 (55)
Tumor type, n (%)										
Renal cell	1 (20)	1 (20)	0	2 (33)	7 (78)	3 (75)	15 (83)	3 (100)	4 (80)	36 (62)
Melanoma with mutation^b^	1 (20)	0	0	0	2 (22)	1 (25)	1 (6)	0	0	5 (9)
Non-squamous NSCLC	2 (40)	2 (40)	0	1 (17)	0	0	0	0	0	5 (9)
Melanoma-unknown	0	0	0	1 (17)	0	0	2 (11)	0	1 (20)	4 (7)
Other^c^	0	1 (20)	2 (67)	0	0	0	0	0	0	3 (5)
Squamous NSCLC	1 (20)	1 (20)	0	0	0	0	0	0	0	2 (3)
Bladder	0	0	1 (33)	0	0	0	0	0	0	1 (2)
Ovarian	0	0	0	1 (17)	0	0	0	0	0	1 (2)
Prostate	0	0	0	1 (17)	0	0	0	0	0	1 (2)
Prior anti-cancer therapy, n (%)^a^										
Biologic	4 (80)	0	0	0	1 (11)	1 (25)	0	1 (33)	1 (20)	8 (14)
Immunotherapy^de^	1 (20)	0	0	1 (17)	0	0	0	0	0	2 (4)
Chemotherapy	5 (100)	4 (80)	3 (100)	3 (50)	0	1 (25)	7 (39)	0	0	23 (40)
Surgery	2 (40)	3 (60)	2 (67)	4 (67)	5 (56)	2 (50)	15 (83)	2 (67)	4 (80)	39 (67)
Radiation	3 (60)	1 (20)	1 (33)	0	3 (33)	3 (75)	9 (50)	2 (67)	2 (40)	24 (41)
Other	3 (60)	1 (20)	2 (67)	4 (67)	5 (56)	2 (50)	9 (50)	3 (100)	3 (60)	32 (55)

*Abbreviations*: *ECOG* Eastern Cooperative Oncology Group, *Fc* fragment crystallizable, *NSCLC* non-small cell lung cancer, *PD-L2* programmed cell death ligand-2 ^a^Data unavailable for 1 patient ^b^All tumors harboring mutations had *BRAF* mutations except for 1 with *EGFR* mutation ^c^Includes adenoma of unknown primary, cellular uterine leiomyoma, and fallopian tube carcinoma ^d^Includes 1 patient enrolled before the May 2014 amendment who received prior AMP-224 PD-L2 Fc fusion protein and 1 patient who received prior pegylated interferon alfa-2b, recorded as an immunotherapy by the investigator ^e^Includes 1 patient who received the therapeutic anticancer vaccine, rocapuldencel-T, plus sunitinib

The study design is shown in Additional file 1: Figure S1a, including dose levels and administration frequency for each dose cohort. Eligible patients had advanced solid malignancies that were refractory to standard therapy or for which no standard therapy existed. They were enrolled if they had ≥1 measurable lesion according to Response Evaluation Criteria in Solid Tumors (RECIST v1.1), had not received previous anti-PD-1/PD-L1 antibodies (expanded in a protocol amendment in May 2014 to exclude any immunotherapy except therapeutic cancer vaccines), had sufficient organ function, and had an Eastern Cooperative Oncology Group (ECOG) performance score of 0 or 1.

Based on accumulating evidence of response to PD-1 inhibition in kidney cancer and melanoma [31–35], the study protocol was amended to enroll only patients with these tumor types in cohorts 5–9. Therefore, the majority of patients had kidney cancer (62%) or melanoma (16%).

Patients received MEDI0680 for 12 months or until progressive disease; those maintaining disease control were followed for an additional 12 months. All patients were followed long-term for survival. Retreatment was permitted in cases of progression during the 12-month follow-up period.

### Endpoints and assessments

#### Primary

The primary endpoint was safety, assessed by evaluating dose-limiting toxicities (DLTs), adverse events (AEs), serious adverse events (SAEs), laboratory evaluations, vital signs, physical examinations, and electrocardiograms. The National Cancer Institute Common Terminology Criteria for Adverse Events Version 4.03 was used to classify and grade AEs and SAEs. Laboratory abnormalities were monitored from the start of the study until 12 months after the last dose of study drug, or until the patient withdrew from follow-up.

Adverse events of special interest (AESIs) included AEs of hepatic function abnormality meeting the definition of Hy’s law, Grade ≥ 3 endocrinopathies, Grade ≥ 3 dermatologic AEs, Grade ≥ 3 pneumonitis, and other Grade ≥ 3 immune-related AEs.

#### Secondary endpoints

The secondary endpoints of this study are shown in Additional file 1: Figure S1b and included assessment of the pharmacokinetics and immunogenicity of MEDI0680, as well as its efficacy.

#### MEDI0680 concentrations in patient serum

The serum concentration of MEDI0680 was determined using a validated electrochemiluminescence (ECL) ligand binding assay format. Standards, controls, and test samples were incubated with biotinylated anti-MEDI0680 bound to a streptavidin-coated plate. Following incubation, ruthenylated anti-IgG4 was added to the plate to allow the formation of molecular complexes. Unbound material was removed by washing the plate, adding MSD read buffer, and detecting bound complexes by ECL using a SECTOR 6000 MSD imager (MesoScale Discovery). Data were analyzed by linear regression using Watson LIMS™ software (Thermo Fisher Scientific) and the concentrations of MEDI0680 in serum were interpolated from a standard curve. The assay lower limit of quantitation was determined to be 0.5 μg/mL and the upper limit of quantitation was 100 μg/mL.

#### Anti-drug antibody responses

Anti-drug antibodies (ADAs) in serum samples were assessed using validated bridging format ECL assays. For all assays, the samples were diluted 1:10 in assay diluent and then incubated with biotinylated and ruthenylated MEDI0680 to allow the formation of molecular complexes. The negative control was a human serum pool, and positive control samples were prepared by spiking the negative control serum pool with ADA. The complexed samples were loaded into wells of a blocked, streptavidin-coated MSD plate, washed, and the bound complexes detected by ECL using a SECTOR 6000 MSD imager (MesoScale Discovery). The data were processed using Watson LIMS™ software (Thermo Fisher Scientific) and the presence of ADAs was determined based on an assay-specific cut-point. Samples that screened as ADA-positive were further assessed using confirmatory and titer assays.

#### Efficacy

The clinical efficacy and antitumor activity secondary endpoints included objective response (OR) and disease control (DC) based on RECIST v1.1 guidelines, modified to require confirmation of progressive disease by a repeat, consecutive assessment no less than 4 weeks from the date of first documentation. The rationale for this modification was to discourage premature discontinuation of the investigational agent and provide a more complete evaluation of its antitumor activity than would be seen with conventional RECIST criteria. Additional secondary endpoints assessed were duration of response (DOR), progression-free survival (PFS), and overall survival (OS).

### Exploratory endpoints

Exploratory endpoints including PD-1 receptor occupancy and the pharmacodynamic profile of MEDI0680 were evaluated to assess the biological activity of the drug in both peripheral blood and tumor biopsy samples (Additional file 1: Figure S1b and Table S2).

#### PD-1 receptor occupancy

Occupancy of the PD-1 receptor by MEDI0680 was determined using a whole blood drug saturation assay. Briefly, potassium EDTA anti-coagulated whole blood samples from study patients were washed and then incubated with formalin buffer or with a saturating dose of MEDI0680 (30 μg/mL) at ambient temperature for 30 min. Bound MEDI0680 was detected using a biotin-labeled anti-human IgG4 antibody followed by Phycoerythrin (PE)-conjugated streptavidin, after washes in between binding steps. Fluorochrome labeled anti-human CD3 and CD45RO antibodies were used to determine PD-1 receptor occupancy on antigen-experienced (CD45RO+) CD3+ T cells. Receptor occupancy was defined as the percentage of CD3+ CD45RO+ cells bound to MEDI0680 after incubation with formulation buffer divided by the percentage of MEDI0680 bound CD3+ CD45RO+ cells after MEDI0680 saturation.

#### T-cell activation and proliferation markers

Peripheral blood mononuclear cell (PBMC) samples were cryopreserved and subsequently evaluated in batches by flow cytometry (BD LSR Fortessa; BD Biosciences). Monoclonal antibodies and viability dye used for flow cytometry panels included: Anti-CD3 BV605, clone SK7 (BD Biosciences); Anti-CD4 PerCP-eFlour710, clone SK3 (eBioscience); Anti-CD8 FITC, clone SK1 (Biolegend); Anti-CCR7 APC, clone G043H7 (Biolegend); Anti-CD45RA PE-Cy7, clone HIT100 (Biolegend); Anti-CD38 BV421, clone HIT2 (Biolegend); Anti-human leukocyte antigen (HLA)-DR PE antibody, clone L243 (Biolegend); Anti-Ki67 BV421, clone B56 (BD Biosciences); Mouse IgG1 BV421, clone X40 (BD Biosciences); Mouse IgG1 PE, clone MOPC21 (Biolegend); Zombie Near-IR Fixable dye (Biolegend). Surface marker staining was followed by intracellular marker staining after fixation and permeabilization. CD4+ and CD8+ T cells were identified after gating on live (Zombie fixable dye negative) CD3+ cells, and CD4 effector memory (T_EM_) cells were defined as CD3 and CD4 double positive cells that were CCR7– and CD45RA–. Levels of the activation markers CD38 and HLA-DR, as well as the intracellular proliferation marker Ki67, were determined on CD4+ and CD8+ T-cell subsets using FlowJo® Software (FlowJo LLC) by setting gates based on a mouse IgG1 isotype control panel.

#### Circulating cytokines

Plasma samples were assessed for levels of the cytokine IFNγ and the chemokines CXCL9 (monokine induced by IFNγ, MIG), CXCL10 (IFNγ-induced protein-10, IP-10), and CXCL11 (interferon-inducible T-cell alpha chemoattractant, I-TAC) using a custom human MULTI-SPOT cytokine 4-plex assay kit and an SI6000 MSD reader (MesoScale Discovery). Sample signals were compared to calibration curves to determine the concentration of each analyte in plasma samples.

#### PD-L1 and CD8 immunohistochemistry

Tumor biopsies were collected prior to treatment and during treatment (cycle 2 between day 1 and day 15); in addition, archival biopsies were assessed when available. The PD-L1 status of tumor samples was determined from 22 evaluable pretreatment fresh (*n* = 21) or archival (*n* = 1) tumor biopsies formaldehyde fixed paraffin-embedded (FFPE) using the VENTANA PD-L1 (SP263) immunohistochemistry (IHC) assay [7]. Samples were classified as having PD-L1 membrane staining of any intensity in ≥ 25% of tumor cells or < 25% of tumor cells [36]. Immunohistochemical staining for CD8 was performed on 14 evaluable fresh paired pre- and on-treatment tumor biopsies (cycle 2 between day 1 and day 15) using rabbit anti-human CD8 monoclonal antibody clone SP239 (Spring Bioscience). Images of immunostained slides were captured using an Aperio digital pathology slide scanner (Leica Biosystems) and examined at 20× magnification. The numbers of CD8+ lymphocytes per entire tissue field containing tumor were counted manually, with a minimum of 3 and a maximum number of 10 fields of view (FOV) counted per case. Areas of necrosis or tissue artifact were excluded. A 20× Aperio image FOV represents 0.4 mm^2^; therefore, mean CD8+ tumor infiltrated lymphocyte (TILs) per mm^2^ were calculated by multiplying the mean number of CD8+ T cells/FOV by 2.5. Non-evaluable specimens were defined as biopsies that did not contain at least 100 tumor cells or specimens that did not maintain adherence to slides during the IHC process.

#### Tumor gene expression

Total RNA was isolated from 11 available and evaluable fresh frozen tumor biopsy samples collected pre- and on-treatment (cycle 2 between day 1 and day 15 in the Q2W or Q3W dosing schedule). The level of RNA transcripts for 171 immune-related genes was measured by TaqMan real-time polymerase chain reaction (Thermo Fisher Scientific) using Fluidigm BioMark 96.96 Dynamic Array chips (Fluidigm Corp). Delta-delta cycle thresholds (ΔΔCt) were calculated for each pre- and on-treatment sample pair and shown as Log2 fold change.

### Statistical analyses

Maximum tolerated dose (MTD) evaluation was based on the DLT-evaluable population, defined as patients who received the protocol-assigned treatment and completed the DLT evaluation period (≥ 21 days for the Q3W schedule and ≥ 28 days for the other dosing schedules) or experienced a DLT during this period. Non-evaluable patients in the dose-escalation phase could be replaced. Tolerability and clinical activity evaluations were based on the as-treated population (all patients receiving any dose of study drug).

For clinical activity, OR was defined as confirmed complete response (CR) or partial response (PR), and DC was defined as CR, PR, or stable disease (SD) for ≥ 24 weeks (DCR24). The objective response rate (ORR) and disease control rate (DCR) were calculated as a percentage of the as-treated population.

## Results

### Preclinical characterization of MEDI0680

MEDI0680 is a humanized mAb of the IgG4 isotype containing a serine-to-proline amino acid replacement in the immunoglobulin fragment crystallizable (Fc) hinge region to stabilize the immunoglobulin and prevent inter-strand fragment antigen-binding (Fab) arm exchange [37]. The mAb bound to PD-1 on activated human T cells with a mean apparent half-maximal (EC_50_) binding value (reflecting bivalent binding) of 822 ± 220 pM (Additional file 1: Figure S2a). The dissociation rate constant (K_D_) for binding of the antibody to recombinant human PD-1 (monovalent binding) was measured as 29 nM by surface plasmon resonance (Biacore). This binding was highly specific for PD-1 as MEDI0680 bound poorly to closely related family members (Additional file 1: Figure S2b and Table S3). The antibody blocked the binding of recombinant human PD-L1 and PD-L2 to human PD-1 expressing Chinese hamster ovary (CHO) cells, with half-maximal inhibitory concentration (IC_50_) values of 2.6 nM and 3.6 nM for PD-L1 and PD-L2, respectively (Additional file 1: Figure S3). Consistent with its PD-1:PD-L1 ligand blocking activity, MEDI0680 enhanced in-vitro IFNγ production in allogeneic dendritic cell / T cell mixed lymphocyte reactions (Additional file 1: Figure S4a) and killing of Epstein–Barr virus (EBV)-expressing esophageal squamous cell carcinoma tumor cells by EBV-reactive primary human T cells (Additional file 1: Figure S4b). The activity of MEDI0680 in these preclinical assays supported testing of the drug in this FTIH phase I clinical trial.

### Patient characteristics

From December 2013 to August 2015, a total of 58 eligible patients with solid tumors were enrolled and treated. Data were collected through November 7, 2017. Patient baseline characteristics are summarized in Table 1 and patient disposition is shown in Additional file 1: Table S4.

Five of the 9 patients with melanoma had tumors bearing known mutations (4 with *BRAF* mutations and 1 with *EGFR* mutation).

### Safety

An MTD was not reached, thus the highest protocol-defined dose was 20 mg/kg Q2W. Treatment-related AEs occurred in 48 patients (83%) across all cohorts (Table 2). The most commonly reported (> 10%) treatment-related AEs of any grade in the study were fatigue (21%), nausea (16%), decreased appetite (16%), vomiting (14%), anemia (12%), pyrexia (12%), arthralgia (12%), pruritus (10%), and asthenia (10%) (Table 3). Grade 1 or 2 treatment-related AEs occurred in 36/58 (62%) patients. Grade 3 or 4 treatment-related AEs occurred in 12/58 (21%) patients; the most common were anemia (4 patients [7%]); fatigue and aspartate aminotransferase increase (each in 2 patients [3%]); and abdominal pain, alanine aminotransferase (ALT) increase, arthralgia, asthenia, autoimmune hepatitis, blood alkaline phosphatase increase, blood creatine phosphokinase increase, dehydration, diarrhea, hypercalcemia, hyperkalemia, hypertension, lipase increase, myasthenia gravis, myositis, and urinary tract infection (each in 1 patient [2%]). Four patients (7%) discontinued due to treatment-related AEs: 1 due to grade 2 pyrexia; 1 due to grade 3 elevated ALT; 1 due to grade 1 creatinine increase, grade 1 potassium increase, grade 3 fatigue, and grade 2 myalgia; and 1 due to grade 2 asthenia. No treatment-related deaths were observed.

**Table 2:** Safety summary in the as-treated population

Event, n (%)^a^	Q3W (mg/kg)					Q2W (mg/kg)		QW × 2 (mg/kg)	QW × 4 (mg/kg)	Total
	Cohort 1 0.1 *n* = 5 (%)	Cohort 2 0.5 *n* = 5 (%)	Cohort 3 2.5 *n* = 3 (%)	Cohort 4 10 *n* = 6 (%)	Cohort 5 20 *n* = 9 (%)	Cohort 6 10 *n* = 4 (%)	Cohort 7 20 *n* = 18 (%)	Cohort 8 20 *n* = 3 (%)	Cohort 9 20 *n* = 5 (%)	*N* = 58 (%)
Any AE	5 (100)	5 (100)	3 (100)	6 (100)	9 (100)	4 (100)	18 (100)	3 (100)	5 (100)	58 (100)
Any grade ≥ 3 AE	2 (40)	5 (100)	1 (33)	5 (83)	6 (67)	2 (50)	11 (61)	1 (33)	1 (20)	34 (59)
Any death (grade 5 AE)	1 (20)	0	0	1 (17)	0	0	2 (11)	0	0	4 (7)
Serious AE	2 (40)	4 (80)	0	3 (50)	5 (56)	1 (25)	10 (56)	1 (33)	2 (40)	28 (48)
AE leading to discontinuation	1 (20)	2 (40)	0	1 (17)	2 (22)	0	2 (11)	0	0	8 (14)
Treatment-related AE	5 (100)	4 (80)	3 (100)	5 (83)	5 (56)	4 (100)	15 (83)	3 (100)	4 (80)	48 (83)
Treatment-related grade ≥ 3 AE	0	3 (60)	0	0	2 (22)	0	6 (33)	0	1 (20)	12 (21)
Treatment-related death	0	0	0	0	0	0	0	0	0	0
Treatment-related serious AE	0	1 (20)	0	1 (17)	1 (11)	1 (25)	3 (17)	0	1 (20)	8 (14)
Treatment-related AE leading to discontinuation	0	2 (40)	0	1 (17)	1 (11)	0	0	0	0	4 (7)

*Abbreviation*: *AE* adverse event

^a^Patients were counted once for each category regardless of the number of events

**Table 3:** Any-grade treatment-related AEs occurring in ≥10% of total population and all grade ≥ 3 treatment-related AEs

	Q3W (mg/kg)					Q2W (mg/kg)		QW × 2 (mg/kg)	QW × 4 (mg/kg)	Total
	Cohort 1 0.1 (*n* = 5)	Cohort 2 0.5 (*n* = 5)	Cohort 3 2.5 (*n* = 3)	Cohort 4 10 (*n* = 6)	Cohort 5 20 (*n* = 9)	Cohort 6 10 (*n* = 4)	Cohort 7 20 (*n* = 18)	Cohort 8 20 (*n* = 3)	Cohort 9 20 (*n* = 5)	*N* = 58
Any-grade treatment-related AE^a^	5 (100)	4 (80)	3 (100)	5 (83)	5 (56)	4 (100)	15 (83)	3 (100)	4 (80)	48 (83)
Fatigue	0	1 (20)	0	1 (17)	1 (11)	1 (25)	7 (39)	1 (33)	0	12 (21)
Decreased appetite	2 (40)	1 (20)	2 (67)	1 (17)	0	0	3 (17)	0	0	9 (16)
Nausea	1 (20)	2 (40)	0	1 (17)	1 (11)	1 (25)	2 (11)	1 (33)	0	9 (16)
Vomiting	2 (40)	1 (20)	0	0	0	1 (25)	3 (17)	1 (33)	0	8 (14)
Anemia	0	1 (20)	0	0	1 (11)	0	3 (17)	0	2 (40)	7 (12)
Arthralgia	0	0	0	0	1 (11)	0	3 (17)	1 (33)	2 (40)	7 (12)
Pyrexia	1 (20)	1 (20)	0	1 (17)	0	1 (25)	2 (11)	0	1 (20)	7 (12)
Asthenia	0	1 (20)	0	0	1 (11)	0	4 (22)	0	0	6 (10)
Pruritus	1 (20)	0	1 (33)	0	1 (11)	0	3 (17)	0	0	6 (10)
Grade ≥ 3 treatment-related AEs^a^	0	3 (60)	0	0	2 (22)	0	6 (33)	0	1 (20)	12 (20)
Anemia	0	1 (20)	0	0	1 (11)	0	1 (6)	0	1 (20)	4 (7)
Fatigue	0	1 (20)	0	0	1 (11)	0	0	0	0	2 (3)
Aspartate aminotransferase increased	0	1 (20)	0	0	0	0	1 (6)	0	0	2 (3)
Asthenia	0	0	0	0	1 (11)	0	0	0	0	1 (2)
Abdominal pain	0	0	0	0	0	0	1 (6)	0	0	1 (2)
Diarrhea	0	0	0	0	0	0	1 (6)	0	0	1 (2)
Autoimmune hepatitis	0	1 (20)	0	0	0	0	0	0	0	1 (2)
Urinary tract infection	0	0	0	0	0	0	1 (6)	0	0	1 (2)
Alanine aminotransferase increased	0	1 (20)	0	0	0	0	0	0	0	1 (2)
Blood alkaline phosphatase increased	0	1 (20)	0	0	0	0	0	0	0	1 (2)
Blood creatine phosphokinase increased	0	0	0	0	0	0	1 (6)	0	0	1 (2)
Lipase increased	0	0	0	0	1 (11)	0	0	0	0	1 (2)
Dehydration	0	0	0	0	1 (11)	0	0	0	0	1 (2)
Hypercalcemia	0	0	0	0	1 (11)	0	0	0	0	1 (2)
Hyperkalemia	0	0	0	0	0	0	1 (6)	0	0	1 (2)
Arthralgia	0	0	0	0	0	0	1 (6)	0	0	1 (2)
Myositis	0	0	0	0	0	0	1 (6)	0	0	1 (2)
Myasthenia gravis	0	0	0	0	0	0	1 (6)	0	0	1 (2)
Hypertension	0	0	0	0	0	0	1 (6)	0	0	1 (2)

All grade ≥ 3 events were grade 3, with the exception of two grade 4 events (one event of increased blood creatine phosphokinase and one event of myositis)

*Abbreviation*: *AE* adverse event

^a^Patients were counted once for each category regardless of the number of events

#### AEs of special interest

Grade 3 treatment-related AESIs occurred in 4/58 patients (7%): ALT and AST increases and autoimmune hepatitis (*n* = 1, discontinued treatment as described above); lipase increase (*n* = 1, resolved); AST increase and myasthenia gravis (*n* = 1, both resolved); and diarrhea (*n* = 1, resolved; no report of colitis). There were no Grade 4 or 5 treatment-related AESIs. Pneumonitis was not observed.

### Clinical activity

**Fig. 1:**
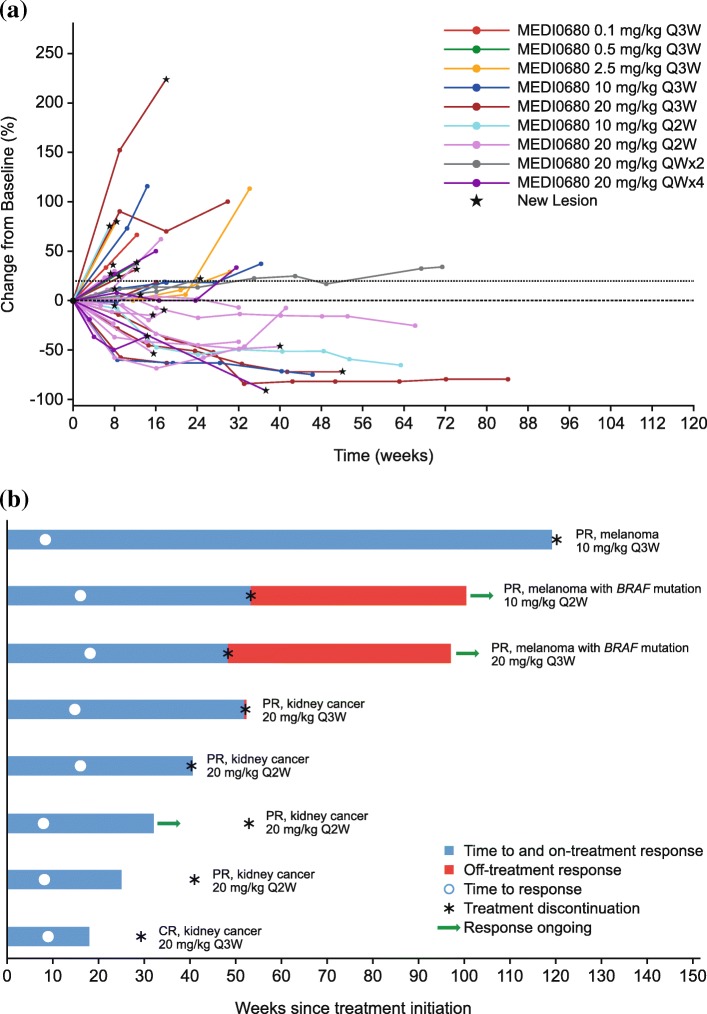
Response to MEDI0680 therapy. **a** Tumor size change from baseline in the as-treated population. **b** The timing and duration of response and onset of progressive disease or new lesions in the responding population. Blue bars indicate treatment initiation to censoring date or progression date. One patient with kidney cancer and PR had an ongoing response but did not have a disease assessment at the time of the last dose

The best objective responses at each dose level are shown in Table 4. In total, 8/58 patients in the as-treated population (14%) had a confirmed OR: 3 had melanoma (2 with *BRAF* mutations) and 5 had kidney cancer, including 1 who had a CR. The DCR24 was 17/58 (29%). Tumor size change from baseline (spider plot) is shown in Fig. 1a. The timing and duration of response and onset of progressive disease or new lesions in the responding population (swimmer’s plot) is shown in Fig. 1b. The DOR ranged from 9.1 to 110.7 weeks. Three of the 8 responders discontinued study treatment without completing the protocol-defined 12 months of treatment; all had kidney cancer. Of these, 1 with CR and 1 with PR discontinued treatment due to progressive disease, and 1 with PR discontinued due to new brain metastases.

**Table 4:** Best overall response in the as-treated population

Response, n (%)	Q3W (mg/kg)					Q2W (mg/kg)		QW x 2 (mg/kg)	QW x 4 (mg/kg)	Total
	Cohort 1 0.1 *n* = 5 (%)	Cohort 2 0.5 *n* = 5 (%)	Cohort 3 2.5 *n* = 3 (%)	Cohort 4 10 *n* = 6 (%)	Cohort 5 20 *n* = 9 (%)	Cohort 6 10 *n* = 4 (%)	Cohort 7 20 *n* = 18 (%)	Cohort 8 20 *n* = 3 (%)	Cohort 9 20 *n* = 5 (%)	*N* = 58 (%)
Objective response	0	0	0	1 (17)	3 (33)	1 (25)	3 (17)	0	0	8 (14)
CR	0	0	0	0	1 (11)^b^	0	0	0	0	1 (2)
PR	0	0	0	1 (17)^c^	2 (22)^d^	1 (25)^e^	3 (17)^b^	0	0	7 (12)
SD	0	0	1 (33)	2 (33)	2 (22)	1 (25)	6 (33)	2 (67)	4 (80)	18 (31)
PD	3 (60)	3 (60)	1 (33)	1 (17)	3 (33)	2 (50)	5 (28)	1 (33)	1 (20)	20 (35)
Non-evaluable^a^	2 (40)	2 (40)	1 (33)	2 (33)	1 (11)	0	4 (22)	0	0	12 (21)

*Abbreviations*: *CR* complete response, *PD* progressive disease, *PR* partial response, *SD* stable disease

^a^Includes patients who discontinued study before first disease assessment and patients for whom not all target lesions were evaluated

^b^Kidney cancer

^c^Melanoma

^d^Kidney cancer (*n* = 1), melanoma with *BRAF* mutation (*n* = 1)

^e^Melanoma with *BRAF* mutation

All 8 responders were in the 10 mg/kg or 20 mg/kg dose cohorts, where peripheral PD-1 receptor occupancy and blood drug concentrations reached a plateau (see pharmacokinetic and receptor occupancy results below). In these pooled 10 and 20 mg/kg cohorts, the ORR was 8/45 (18%); all responses were observed in the Q2W and Q3W groups. The DCR24 was 17/45 (37%) in this subset.

Two patients entered retreatment after the initial 12-month period (1 receiving 10 mg/kg Q3W and the other receiving 20 mg/kg Q2W), but discontinued due to progressive disease.

### MEDI0680 pharmacokinetic and pharmacodynamic profiles

#### Pharmacokinetics, ADA responses, and PD-1 receptor occupancy

**Fig. 2:**
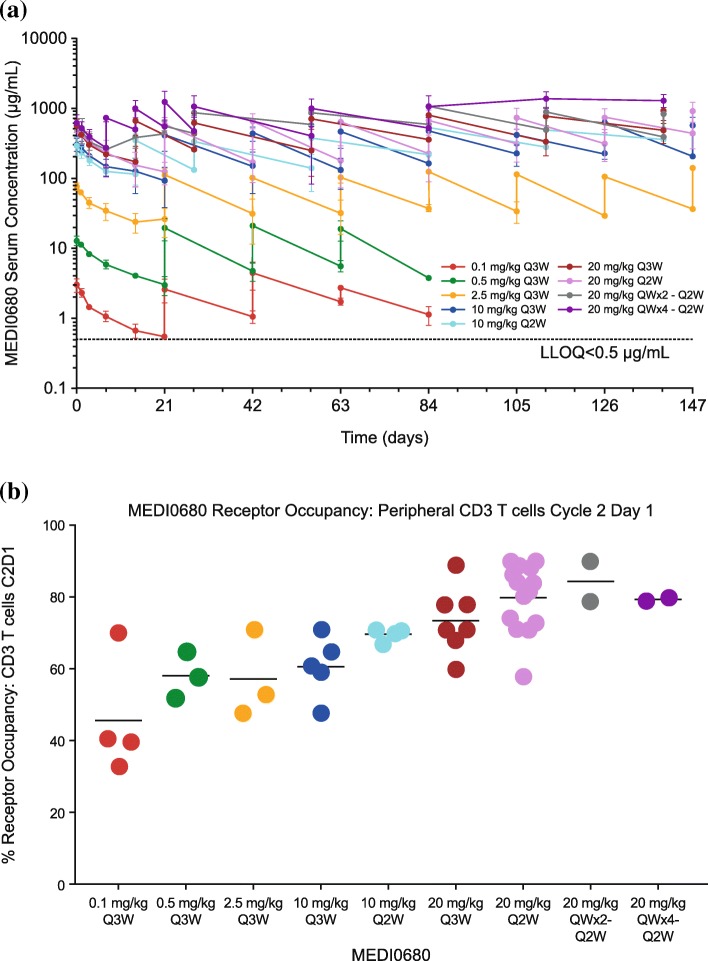
Pharmacokinetic and receptor occupancy analysis of MEDI0680. **a** Pharmacokinetic analysis of MEDI0680 in patient serum. Data represent time points up to 150 days. Abbreviation: *LLOQ* lower limit of quantitation. **b** PD-1 receptor occupancy by MEDI0680 on CD45 RO+ CD3 T cells among patients treated at various drug doses and schedules, as indicated. Measurements were done at baseline, during the first cycle of MEDI0680 treatment, and on the first day after the completion of the first cycle

A dose-proportional increase in peak serum MEDI0680 concentration was observed (Fig. 2a). The mean terminal half-life was estimated to be 19 days, with a standard deviation of 5.6 days at 20 mg/kg Q2W dosing based on simulations in a MEDI0680 population pharmacokinetics model (*n* = 1000) [38]. Fifty-four patients were evaluated for the development of post-baseline ADAs and 8 (15%) tested positive post-dose. Based on samples from 40 patients, a dose-dependent saturation of PD-1 was observed on CD3+ T cells, with median PD-1 receptor occupancy ≥70% after 1 cycle of MEDI0680 treatment at 10 or 20 mg/kg; the highest, most consistent occupancy was obtained with initial weekly dosing at 20 mg/kg (Fig. 2b).

#### T-cell activation and proliferation, and cytokine levels in peripheral blood

**Fig. 3:**
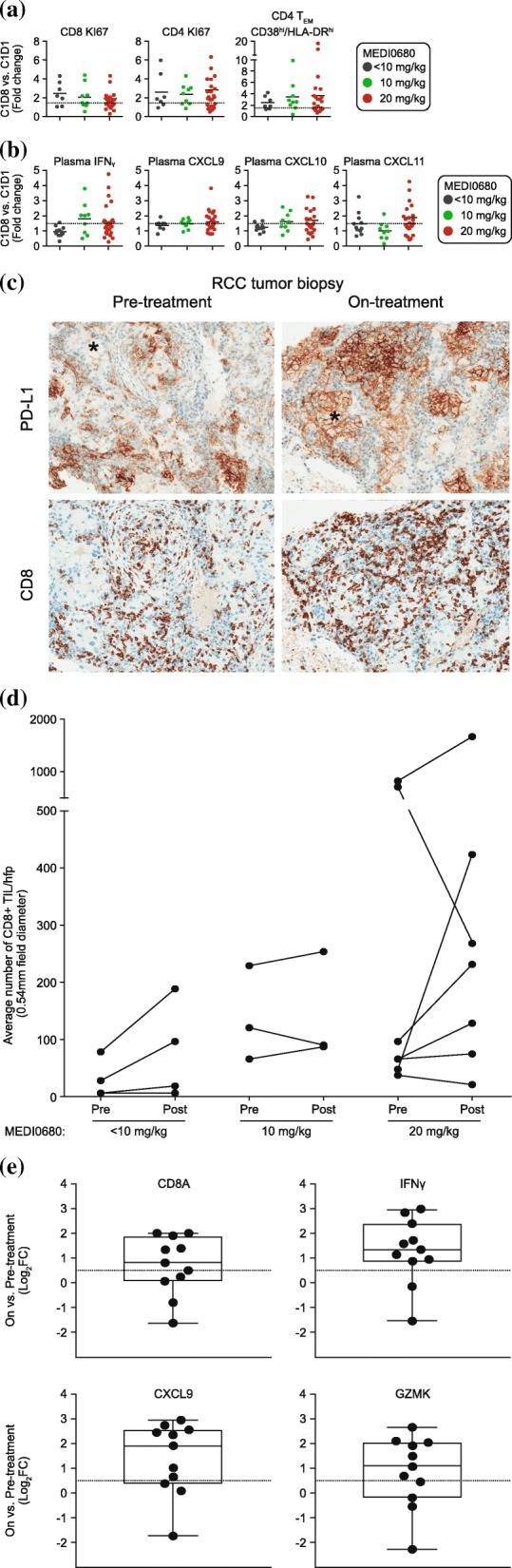
Peripheral and intratumoral measures of MEDI0680 activity. **a** Peripheral CD4+ and CD8+ T-cell activation and proliferation among treatment groups, as indicated. Shown are the fold changes in the percentages of CD4+ and CD8+ Ki67+ and CD4+ T_EM_ CD38^high^/HLA-DR^high^ cells in whole blood post-treatment. Abbreviation: *T*_*EM*_ effector memory T cells. **b** Change in plasma cytokines among treatment groups, as indicated. Shown are the fold change in the plasma levels of IFNγ, CXCL-9, CXCL-10, and CXCL-11 at day 8 post-treatment with MEDI0680. **c** Examples of PD-L1+ and CD8+ IHC images (20× magnification) from matched pre- and on-treatment biopsies from an RCC patient. The tumor at screening is characterized by abundant CD8+ TILs and PD-L1 on immune cells but not on tumor cells (* symbols on IHC images). The on-treatment tumor has greater CD8+ T-cell infiltration and PD-L1 immunoreactivity on both immune and tumor cells (*). **d** Levels of CD8+ TILs in tumor biopsies pre- and on-treatment at various dose levels. Abbreviation: *hpf* high power field. (**e**) Log_2_ fold change in on-treatment versus pretreatment *CD8A*, *IFNG*, *CXCL9*, and *GZMK* gene expression in RCC and melanoma tumor biopsies. A 1.5-fold change is indicated by the dotted line

Among total CD4+ and CD8+ T cells from patients receiving < 10 mg/kg, 10 mg/kg or 20 mg/kg, at least a 2-fold median increase in the percentage of Ki67+ T cells and activated CD38^high^/HLA-DR^high^ CD4+ T_EM_ cells was observed on day 8 post-treatment during the first cycle (Fig. 3a and Additional file 1: Figure S5). Consistent with MEDI0680-dependent peripheral T-cell activation, plasma levels of IFNγ and CXCL9 (MIG), CXCL10 (IP-10), and CXCL11 (I-TAC) were increased on-treatment with a median 1.5-fold change among patients receiving 10 or 20 mg/kg MEDI0680, with the exception of CXCL11 in patients dosed within the 10 mg/kg cohorts, where no on-treatment median change was observed (Fig. 3b). There was no correlation between increased peripheral biomarkers and clinical response at any MEDI0680 dose level (Additional file 1: Figure S6).

#### PD-L1 expression and T-cell density and activation in tumor biopsies

Among 22 evaluable pretreatment tumor biopsies, 2/22 (9.1%) were scored PD-L1 ≥ 25% and 20/22 (91%) were PD-L1 < 25%. None of the responders had evaluable tissue for PD-L1 staining. The PD-L1 ≥ 25% biopsies were from a non-squamous non-small cell lung cancer (NSCLC) patient and a melanoma patient; the former was not evaluable for clinical response and the latter had progressive disease as the best OR. Of the 20 patients with PD-L1 < 25%, 2 were not evaluable for clinical response, 10 had SD, and 8 had progressive disease. CD8+ T-cell density and gene expression were evaluated from 14 paired pre- and on-treatment fresh tumor biopsies to determine MEDI0680 activity. During treatment, 8/14 (57%) samples across all dose cohorts showed a 2-fold or greater increase in intratumoral CD8+ T-cell density as measured by IHC (Fig. 3c and d). This was consistent with an increase in *CD8A* gene expression and genes associated with T-cell effector function (Fig. 3e). On-treatment, 2-fold or greater increases in gene expression of *IFNG*, *CXCL9* (a T-cell chemoattractant), and *GZMK* (a marker of cytolytic T-cell activity) were also observed (Fig. 3e). Although association with clinical response could not be determined due to a small sample size of evaluable tumor biopsies, MEDI0680 treatment did elicit T-cell infiltration and/or expansion and showed pharmacodynamic evidence of immune-related antitumor activity.

## Discussion

In this FTIH phase I study, MEDI0680 had a tolerable safety profile and demonstrated clinical activity. No treatment-related deaths were observed and the majority of treatment-related AEs were mild to moderate (62% grade 1/2 and 21% grade 3/4). Four patients (7%) discontinued MEDI0680 due to treatment-related AEs. An MTD was not reached.

PD-1 receptor occupancy appeared to reach a peak in the 10 mg/kg Q2W and 20 mg/kg Q3W cohorts. Additional patients were recruited at the 20 mg/kg Q2W dose level, including a cohort with weekly dosing for 2 weeks followed by Q2W dosing (20 mg/kg QWx2) and one with weekly dosing for 4 weeks followed by Q2W dosing (20 mg/kg QWx4) to increase confidence that receptor saturation had peaked. No significant differences in receptor occupancy between these groups at cycle 2, day 1 were observed, although median values were numerically higher for the 20 mg/kg cohorts compared to the 10 mg/kg Q2W group. Likewise, pharmacokinetic profiling showed similar trough serum drug levels between the 10 and 20 mg/kg doses, but with numerically higher values for the latter. Considering peripheral PD-1 receptor occupancy and drug levels together with peripheral pharmacodynamic data showing comparable drug activity and similar tolerability profiles at the 10 mg/kg and 20 mg/kg dose levels, 20 mg/kg Q2W was declared the highest protocol-defined dose. Because circulating drug must penetrate tumors against interstitial fluid pressure gradients and despite endocytic consumption within tumors [39], the 20 mg/kg Q2W dose is expected to provide the optimal PD-1 receptor occupancy within tumor tissues themselves.

The safety profile observed in this study was consistent with that of other drugs targeting the PD-1 pathway in patients with solid tumors [40–43]. For example, in a phase I study of 30 patients (with various advanced solid tumors) treated with pembrolizumab 1–10 mg/kg Q2W or 2–10 mg/kg Q3W, 70% of patients had treatment-related AEs (all grade 1 or 2) [27]. In another phase I study of advanced malignancies that included 107 patients with advanced melanoma, Topalian and colleagues showed an 84% incidence of treatment-related AEs with nivolumab 0.1–10 mg/kg Q2W; the events were grade 3/4 in 22% of patients [31]. In the current study, grade 3 treatment-related AESIs occurred in 7% of patients; no grade 4 or 5 AESIs were observed. There were no reports of pneumonitis.

In the current study, confirmed objective clinical responses were seen in 8/58 (14%) patients. All responses were in patients with melanoma or kidney cancer, including a single confirmed CR. A response rate of 8/45 (18%) was seen in patients receiving 10–20 mg/kg. Because the trial comprised 11 tumor types and 9 doses, it is not possible to directly compare the response rates seen with MEDI0680 to those of other PD-1 antibodies. However, the 34 patients with kidney cancer and 8 patients with melanoma treated at the 10–20 mg/kg dose levels indicate the response may be similar to other PD-1 antibodies approved for those indications. A 15% (5/34) response rate was seen in kidney cancer patients receiving 10–20 mg/kg doses. Among patients with advanced/metastatic RCC, nivolumab showed response rates of 27% in a phase I study, 9–22% in a phase Ib study, and 20–22% in a phase II study [43–45]. Pembrolizumab demonstrated an ORR of 34% in a phase II study as first-line treatment in advanced clear cell RCC [27]; this may be numerically higher than response rates seen for nivolumab as well as MEDI0680 in the current study due to the enrollment of patients that had not received prior systemic therapy. In melanoma patients receiving 10–20 mg/kg in this study, MEDI0680 demonstrated a 38% (3/8) response rate. Pembrolizumab showed response rates of 26% and 38% in advanced melanoma in two phase I studies of 173 and 135 patients, respectively [24, 32]. Response rates with nivolumab in advanced melanoma were 28% in the study of solid tumors described above, and 40% in a large phase III randomized trial [31, 33].

As secondary and exploratory endpoints, the pharmacokinetic and pharmacodynamic profiles of MEDI0680 were explored and examined for association with clinical response. Doses of 10–20 mg/kg showed a maximum PD-1 receptor occupancy ≥70%, comparable to the peak occupancy reported for nivolumab [46]. PD-1 targeting by MEDI0680 showed consistent immunological modulation across dose levels, with a frequent increase of activated CD4+ T_EM_ cells (CD38^high^/HLA-DR^high^) and enhanced proliferation (Ki67 positivity) of CD4+ and CD8+ T cells. This is consistent with the induction of peripheral T-cell activation and proliferation markers monitored in other anti-PD-1 clinical trials [46, 47]. In plasma, MEDI0680 increased circulating IFNγ and IFNγ-induced cytokines (CXCL9, 10, and 11) as previously reported [48]. Similar to these findings, Das et al. examined gene transcription changes in isolated peripheral blood T cells from melanoma patients treated with nivolumab and found changes indicative of natural killer (NK) cell expansion and cytolytic function that included upregulation of the *IFNG* transcript [49]. They also found increased serum levels of the IFNγ-inducible cytokine CXCL10; in their study, CXCL9 and CXCL11 were not examined. However, they found neither increased plasma IFNγ cytokine levels nor upregulation of Ki67 transcript or protein in nivolumab-treated patients, as was found in the current study with MEDI0680. This discrepancy could be the result of differences in the time of assessment and/or sensitivity of plasma cytokine assays used. Peripheral biomarker modulation by MEDI0680 was observed in all patients regardless of clinical response, although some had only minimal changes. A lack of correlation between peripheral immune response to PD-1 inhibitors and clinical response has been reported in melanoma patients treated with pembrolizumab [46], although results in NSCLC patients treated with anti-PD-1 therapies suggest that an early versus late induction of immune activation in a specific subpopulation of CD8+ T cells (Ki67+ PD-1+) may enrich for response [47].

In evaluable tumor biopsies, MEDI0680 showed intratumoral pharmacodynamic activity as evidenced by the induction of CD8+ T-cell infiltration and/or expansion and increases of *IFNG* and IFNγ-inducible gene expression consistent with the mechanisms of action of anti-PD-1 blocking agents. Increased CD8+ T-cell infiltration/proliferation, PD-1, PD-L1, granzyme B, and phosphorylated STAT1 positive immune cells in melanoma tumors have been observed by IHC or gene expression after treatment with pembrolizumab or nivolumab in other trials [50, 51]. In these studies, pretreatment or on-treatment levels of T cells within the tumor or at the tumor margin demonstrated predictive value for response to anti-PD-1 therapy [50, 51]. Other trials with PD-1 inhibitors have also identified pretreatment and post-treatment immune cell correlates with response to therapy [52–62]. For example, Daud et al. showed that patients whose melanoma tumors contained ≥ 20% CD8+ T cells with a CTLA-4 high/PD-1 high phenotype demonstrated a significantly higher ORR to anti-PD-1 blockade compared to those whose tumors contained < 20% of these cells [56]. Inoue et al. described higher pretreatment CD8+/Treg and CD8+/CD4+ expression ratios and higher lytic enzyme (*GZMA*) and major histocompatibility complex class I (*HLA-A*) expression correlating with anti-PD-1 mAb response in melanoma [57]. Likewise, others have demonstrated that pretreatment IFNγ-related immune gene signatures predicted response to anti-PD-1 therapy in head and neck squamous cell carcinoma, gastric cancer, and melanoma [48, 49, 51]. Collectively, these findings suggest that upregulation of CD8+ T cells and markers of effector T-cell function are common pharmacodynamic biomarkers of anti-PD-1 blockade, and pre- or post-treatment intratumoral levels in some settings are associated with clinical response.

In conclusion, this study demonstrated that MEDI0680 is a clinically active anti-PD-1 antibody with a tolerable safety profile. Maximum receptor occupancy was achieved at doses where most patients showed evidence of peripheral and intratumoral immune-cell activation. MEDI0680 is currently undergoing clinical testing in combination with the anti-PD-L1 mAb durvalumab versus nivolumab monotherapy in patients with kidney cancer (NCT02118337).

## Additional file

Additional file 1: **Figure S1.** Study design and pharmacokinetic/pharmacodynamic assessment. (**a**) phase I study design. (**b**) Overview of pharmacokinetic and pharmacodynamic profile assessment. **Figure S2**. MEDI0680 binding and specificity for PD-1. (**a**) MEDI0680 binding to activated primary human T cells. (**b**) Binding specificity of MEDI0680 to recombinant human proteins that share amino acid sequence homology with PD-1. **Figure S3.** Inhibition of ligand binding to native PD-1 by MEDI0680. (**a**) Blockade by MEDI0680 of recombinant human PD-L1 or (**b**) recombinant human PD-L2 binding to CHO cells expressing human PD-1 protein. **Figure S4**. In vitro T-cell activation and cytotoxicity mediated by MEDI0680. (**a**) IFNγ release into cell culture media of allogeneic dendritic cell–T cell mixed lymphocyte reactions. (**b**) Cellular cytotoxicity mediated by EBV-reactive CD8 T cells over time, as determined by non-invasive electrical impedance measurement in an xCelligence RTCA MP instrument as a surrogate for cell death. **Figure S5.** Representative examples of flow cytometry of peripheral blood from patients treated with MEDI0680. (**a**) Ki67 staining in CD4+ and CD8+ T cells at cycle 1 day 1 pre-treatment (C1D1) and at cycle 1 day 8 post-treatment, as indicated. (**b**) HLA-DR and CD38 co-staining on CD4+ effector memory T cells (CD4+ TEM) at the same time points. **Figure S6.** Lack of correlation between changes in peripheral pharmacodynamic markers and objective clinical response. (**a**) Fold change in the indicated cytokine and chemokine markers in all cohorts or (**b**) only in the 10 and 20mg/kg cohorts or (c) the fold change in T-cell proliferation and CD4+ TEM CD38high HLA-DRhigh (activated) T cells with respect to objective clinical responses are shown. **Table S1.** Key eligibility criteria. **Table S2.** Patient characteristics and samples evaluated for pharmacodynamic analysis. **Table S3.** In silico identification of PD-1 paralogs using the protein Basic Local Alignment Search Tool BLASTp. **Table S4.** Study disposition (as-treated population). (ZIP 5.02 mb)

## Data Availability

The clinical dataset analyzed during the current study is available at clinicaltrials.gov, https://clinicaltrials.gov/ct2/show/results/NCT02013804. Other datasets used and/or analyzed during the current study are available and may be obtained in accordance with AstraZeneca’s data sharing policy, which is described at https://astrazenecagrouptrials.pharmacm.com/ST/Submission/Disclosure.

## Abbreviations

Ab: Antibody
ADA: Anti-drug antibody
AE: Adverse event
ALT: Alanine aminotransferase
ANOVA: Analysis of variance
AUC: Area under the curve
BOR: Best overall response
CDR: Complementarity-determining region
CHO: Chinese hamster ovary
CR: Complete response
DC: Disease control
DCR: Disease control rate
DCR24: Disease control rate ≥ 24 weeks
DLT: Dose-limiting toxicity
DOR: Duration of response
E:T: Effector-to-target
EBV: Epstein–Barr virus
EC_50_: Drug concentration giving half-maximal response
ECACC: European Collection of Authenticated Cell Cultures
ECL: Electrochemiluminescence
ECOG: Eastern Cooperative Oncology Group
EDTA: Ethylenediaminetetraacetic acid
ELISA: Enzyme-linked immunosorbent assay
Fab: Fragment antigen-binding
FC: Fold change
Fc: Fragment crystallizable
FFPE: Formaldehyde fixed paraffin-embedded
FOV: Fields of view
FTIH: First-time-in-human
GM-CSF: Granulocyte-macrophage colony-stimulating factor
HLA: Human leukocyte antigen
IC_50_: Inhibitor concentration where the response (or binding) is reduced by half
IFNγ: Interferon gamma
IgG: Immunoglobulin G
IHC: Immunohistochemistry
IL-4: Interleukin-4
IP-10: Interferon gamma-induced protein-10
I-TAC: Interferon-inducible T-cell alpha chemoattractant
K_D_: Dissociation rate constant
mAbs: Monoclonal antibodies
MdFI: Median fluorescence intensity
MFI: Mean fluorescence intensity
MTD: Maximum tolerated dose
NK: Natural killer
NSCLC: Non-small cell lung cancer
OR: Objective response
ORR: Objective response rate
OS: Overall survival
PBMC: Peripheral blood mononuclear cell
PCR: Polymerase chain reaction
PD-1: Programmed cell death-1
PD-L1: Programmed cell death ligand-1
PD-L2: Programmed cell death ligand-2
PE: Phycoerythrin
PFS: Progression-free survival
PR: Partial response
Q2W: Every 2 weeks
Q3W: Every 3 weeks
QW: Every week
RCC: Renal cell carcinoma
RECIST: Response Evaluation Criteria in Solid Tumors
SAE: Serious adverse event
SD: Stable disease
SPR: Surface plasmon resonance
T_EM_: T effector memory cells
TIL: Tumor infiltrating lymphocyte
ΔΔCt: Delta-delta cycle thresholds

## References

[1] Dong H, Zhu G, Tamada K, Chen L (1999). B7-H1, a third member of the B7 family, co-stimulates T-cell proliferation and interleukin-10 secretion. Nat Med.

[2] Latchman Y, Wood CR, Chernova T, Chaudhary D, Borde M, Chernova I (2001). PD-L2 is a second ligand for PD-1 and inhibits T cell activation. Nat Immunol.

[3] Pardoll DM (2012). The blockade of immune checkpoints in cancer immunotherapy. Nat Rev Cancer.

[4] Zheng H, Liu X, Zhang J, Rice SJ, Wagman M, Kong Y (2016). Expression of PD-1 on CD4+ T cells in peripheral blood associates with poor clinical outcome in non-small cell lung cancer. Oncotarget..

[5] Goldberg MV, Maris CH, Hipkiss EL, Flies AS, Zhen L, Tuder RM (2007). Role of PD-1 and its ligand, B7-H1, in early fate decisions of CD8 T cells. Blood..

[6] Topalian SL, Drake CG, Pardoll DM (2015). Immune checkpoint blockade: a common denominator approach to cancer therapy. Cancer Cell.

[7] OPDIVO® (nivolumab) US Prescribing Information. Updated November 2018. Available at: https://packageinserts.bms.com/pi/pi_opdivo.pdf. Last Accessed 20 Feb 2019.

[8] KEYTRUDA® (pembrolizumab) US Prescribing Information. Updated December 2018. Available at: https://www.merck.com/product/usa/pi_circulars/k/keytruda/keytruda_pi.pdf. Last Accessed 20 Feb 2019).

[9] Libtayo (cemiplimab) Prescribing Information. Available at: https://www.accessdata.fda.gov/drugsatfda_docs/label/2018/761097s000lbl.pdf. Last Accessed 20 Feb 2019.

[10] Yarchoan M, Hopkins A, Jaffee EM (2017). Tumor mutational burden and response rate to PD-1 inhibition. N Engl J Med.

[11] Lawrence MS, Stojanov P, Polak P, Kryukov GV, Cibulskis K, Sivachenko A (2013). Mutational heterogeneity in cancer and the search for new cancer genes. Nature..

[12] Yi M, Jiao D, Xu H, Liu Q, Zhao W, Han X (2018). Biomarkers for predicting efficacy of PD-1/ PD-L1 inhibitors. Mol Cancer.

[13] Bermejo MB, Jaffee EM, Davar D, Cardarelli J, Williams D, Phillips P. Phase 1b/2 study of nivolumab in combination with an anti–IL-8 monoclonal antibody, BMS-986253, in a biomarker-enriched population of patients with advanced cancer. J Clin Oncol. 2018;36(15_Suppl).

[14] Wrangle JM, Velcheti V, Patel MR, Garrett-Mayer E, Hill EG, Ravenel JG (2018). ALT-803, an IL-15 superagonist, in combination with nivolumab in patients with metastatic non-small cell lung cancer: a non-randomised, open-label, phase 1b trial. Lancet..

[15] Zibelman MR, Macfarlane A, Alpaugh RK, Dulaimi E, Costello K, O’Neill J (2017). Effect of exogenous interferon-gamma (IFN-gamma) on peripheral blood immune markers as part of a phase I clinical trial of combined IFN-gamma with nivolumab (Nivo) in patients (pts) with select solid tumors. J Clin Oncol.

[16] Chung V, Kos FJ, Hardwick N (2019). Evaluation of safety and efficacy of p53MVA vaccine combined with pembrolizumab in patients with advanced solid cancers. Clin Transl Oncol.

[17] Hamid O, Chow LQM, Tavakkoli F, Marshall S, Gribbin MJ, Karakunnel JJ, et al. Phase I, open-label study of MEDI0680, an anti-programmed cell death-1 (PD-1) antibody, in combination with MEDI4736, an anti-programmed cell death ligand-1 (PD-L1) antibody, in patients with advanced malignancies. J Clin Oncol. 2015;33(15_suppl) (Abstract TPS3087).

[18] Janku F, Tan DS, Martin-Liberal J, Takahashi S, Geva R, Gucalp A, et al. First-in-human study of FAZ053, an anti-PD-L1 mAb, alone and in combination with spartalizumab, an anti-PD-1 mAb, in patients with advanced malignancies. J Immunother Cancer. 2018;6(suppl 1) Abstract P651. 10.1016/j.esmoop.2025.105051 PMC12146532 40381383

[19] Butte MJ, Keir ME, Phamduy TB, Sharpe AH, Freeman GJ (2007). Programmed death-1 ligand 1 interacts specifically with the B7-1 costimulatory molecule to inhibit T cell responses. Immunity..

[20] Park JJ, Omiya R, Matsumura Y, Sakoda Y, Kuramasu A, Augustine MM (2010). B7-H1/CD80 interaction is required for the induction and maintenance of peripheral T-cell tolerance. Blood..

[21] Paterson AM, Brown KE, Keir ME, Vanguri VK, Riella LV, Chandraker A (2011). The programmed death-1 ligand 1:B7-1 pathway restrains diabetogenic effector T cells in vivo. J Immunol.

[22] Mazza C, Escudier B, Albiges L (2017). Nivolumab in renal cell carcinoma: latest evidence and clinical potential. Ther Adv Med Oncol.

[23] Weinstock M, McDermott D (2015). Targeting PD-1/PD-L1 in the treatment of metastatic renal cell carcinoma. Ther Adv Urol.

[24] Ivashko IN, Kolesar JM (2016). Pembrolizumab and nivolumab: PD-1 inhibitors for advanced melanoma. Am J Health Syst Pharm.

[25] Galluzzi L, Kroemer G, Eggermont A (2014). Novel immune checkpoint blocker approved for the treatment of advanced melanoma. OncoImmunology..

[26] Brahmer JR, Hammers H, Lipson EJ (2015). Nivolumab: targeting PD-1 to bolster antitumor immunity. Future Oncol.

[27] McDermott David F., Lee Jae-Lyun, Szczylik Cezary, Donskov Frede, Malik Jahangeer, Alekseev Boris Yakovlevich, Larkin James M. G., Matveev Vsevolod Borisovich, Gafanov Rustem Airatovich, Tomczak Piotr, Tykodi Scott S., Geertsen Poul F., Wiechno Pawel J., Shin Sang Joon, Pouliot Frederic, Alonso Gordoa Teresa, Li Wenting, Perini Rodolfo F., Schloss Charles, Atkins Michael B. (2018). Pembrolizumab monotherapy as first-line therapy in advanced clear cell renal cell carcinoma (accRCC): Results from cohort A of KEYNOTE-427. Journal of Clinical Oncology.

[28] Shim H (2011). One target, different effects: a comparison of distinct therapeutic antibodies against the same targets. Exp Mol Med.

[29] Kim MS, Lee SH, Song MY, Yoo TH, Lee BK, Kim YS (2007). Comparative analyses of complex formation and binding sites between human tumor necrosis factor-alpha and its three antagonists elucidate their different neutralizing mechanisms. J Mol Biol.

[30] Emi Aikawa N, de Carvalho JF, Artur Almeida Silva C, Bonfá E (2010). Immunogenicity of anti-TNF-alpha agents in autoimmune diseases. Clin Rev Allergy Immunol.

[31] Topalian SL, Sznol M, McDermott DF, Kluger HM, Carvajal RD, Sharfman WH (2014). Survival, durable tumor remission, and long-term safety in patients with advanced melanoma receiving nivolumab. J Clin Oncol.

[32] Hamid O, Robert C, Daud A, Hodi FS, Hwu WJ, Kefford R (2013). Safety and tumor responses with lambrolizumab (anti-PD-1) in melanoma. N Engl J Med.

[33] Robert C, Long GV, Brady B, Dutriaux C, Maio M, Mortier L (2015). Nivolumab in previously untreated melanoma without BRAF mutation. N Engl J Med.

[34] Patnaik A, Kang SP, Rasco D, Papadopoulos KP, Elassaiss-Schaap J, Beeram M (2015). Phase I study of pembrolizumab (MK-3475; anti-PD-1 monoclonal antibody) in patients with advanced solid tumors. Clin Cancer Res.

[35] Robert C, Ribas A, Wolchok JD, Hodi FS, Hamid O, Kefford R (2014). Anti-programmed-death-receptor-1 treatment with pembrolizumab in ipilimumab-refractory advanced melanoma: a randomised dose-comparison cohort of a phase 1 trial. Lancet..

[36] Rebelatto MC, Midha A, Mistry A, Sabalos C, Schechter N, Li X (2016). Development of a programmed cell death ligand-1 immunohistochemical assay validated for analysis of non-small cell lung cancer and head and neck squamous cell carcinoma. Diagn Pathol.

[37] Silva J-P, Vetterlein O, Jose J, Peters S, Kirby H (2015). The S228P mutation prevents *in vivo* and *in vitro* IgG4 fab-arm exchange as demonstrated using a combination of novel quantitative immunoassays and physiological matrix preparation. J Biol Chem.

[38] Song X, Gao X, Zheng Bo, Black C, Gribbin M, Karakunnel J, et al. Pharmacokinetics and pharmacodynamics of MEDI0680, a fully human anti-PD1 monoclonal antibody, in patients with advanced malignancies. Cancer Res. 2017;77(13 suppl) (Abstract 5045).

[39] Thurber GM, Schmidt MM, Wittrup KD (2008). Antibody tumor penetration: transport opposed by systemic and antigen-mediated clearance. Adv Drug Deliv Rev.

[40] Weber JS, Postow M, Lao CD, Schadendorf D (2016). Management of adverse events following treatment with anti-programmed death-1 agents. Oncologist..

[41] Wang M, Ma X, Guo L, Xia F (2017). Safety and efficacy profile of pembrolizumab in solid cancer: pooled reanalysis based randomized controlled trials. Drug Des Devel Ther.

[42] Wong AC, Ma B (2016). An update on the pharmacodynamics, pharmacokinetics, safety and clinical activity of nivolumab in the treatment of solid cancers. Expert Opin Drug Metab Toxicol.

[43] Topalian SL, Hodi FS, Brahmer JR, Gettinger SN, Smith DC, McDermott DF (2012). Safety, activity, and immune correlates of anti-PD-1 antibody in cancer. N Engl J Med.

[44] Choueiri TK, Fishman MN, Escudier B, McDermott DF, Drake CG, Kluger H (2016). Immunomodulatory activity of nivolumab in metastatic renal cell carcinoma. Clin Cancer Res.

[45] Motzer RJ, Rini BI, McDermott DF, Redman BG, Kuzel TM, Harrison MR (2015). Nivolumab for metastatic renal cell carcinoma: results of a randomized phase II trial. J Clin Oncol.

[46] Huang AC, Postow MA, Orlowski RJ, Mick R, Bengsch B, Manne S (2017). T cell invigoration to tumor burden ratio associated with PD-1 response. Nature..

[47] Kamphorst AO, Pillai RN, Yang S, Nasti TH, Akondy RS, Wieland A (2017). Proliferation of PD-1+ CD8 T cells in peripheral blood after PD-1 targeted therapy in lung cancer patients. Proc Natl Acad Sci U S A.

[48] Murphy KM, Reiner SL (2002). Decision making in the immune system: the lineage decisions of helper T cells. Nat Rev Immunol.

[49] Das R, Verma R, Sznol M, Boddupalli CS, Gettinger SN, Kluger H (2015). Combination therapy with anti-CTLA4 and anti-PD1 leads to distinct immunologic changes in-vivo. J Immunol.

[50] Tumeh PC, Harview CL, Yearly JH, Shintaku IP, Taylor EJ, Robert L (2014). PD-1 blockade induces responses by inhibiting adaptive immune resistance. Nature..

[51] Riaz N, Havel JJ, Makarov V, Desrichard A, Urba WJ, Sims JS (2017). Tumor and microenvironment evolution during immunotherapy with nivolumab. Cell..

[52] Diggs LP, Hsueh EC (2017). Utility of PD-L1 immunohistochemistry assays for predicting PD-1/PD-L1 inhibitor response. Biomark Res.

[53] Festino L, Botti G, Lorigan P, Masucci GV, Hipp JD, Horak CE (2016). Cancer treatment with anti-PD-1/PD-L1 agents: is PD-L1 expression a biomarker for patient selection?. Drugs..

[54] Taube J, Klein A, Brahmer J, Xu H, Pan X, Kim JH (2014). Association of PD-1, PD-1 ligands, and other features of the tumor immune microenvironment with response to anti-PD-1 therapy. Clin Cancer Res.

[55] Chen PL, Roh W, Reuben A, Cooper ZA, Spencer CN, Prieto PA (2016). Analysis of immune signatures in longitudinal tumor samples yields insight into biomarkers of response and mechanisms of resistance to immune checkpoint blockade. Cancer Discov.

[56] Daud A, Loo K, Pauli ML, Sanchez-Rodriguez R, Sandoval PM, Taravati K (2016). Tumor immune profiling predicts response to anti-PD-1 therapy in human melanoma. J Clin Invest.

[57] Inoue H, Park JH, Kiyotani K, Zewde M, Miyashita A, Jinnin M (2016). Intratumoral expression levels of PD-L1, GZMA, and HLA-A along with oligoclonal T cell expansion associate with response to nivolumab in metastatic melanoma. Oncoimmunology..

[58] Seiwert Tanguy Y., Burtness Barbara, Weiss Jared, Eder Joseph Paul, Yearley Jennifer, Murphy Erin, Nebozhyn Michael, McClanahan Terri, Ayers Mark, Lunceford Jared K., Mehra Ranee, Heath Karl, Cheng Jonathan D., Chow Laura Q. (2015). Inflamed-phenotype gene expression signatures to predict benefit from the anti-PD-1 antibody pembrolizumab in PD-L1+ head and neck cancer patients. Journal of Clinical Oncology.

[59] Ayers M, Luceford J, Nebozhyn M, Murphy E, Loboda A, Kaufman DR (2017). IFN-**γ**–related mRNA profile predicts clinical response to PD-1 blockade. J Clin Invest.

[60] Ribas A, Shin DS, Zaretsky J, Frederiksen J, Cornish A, Avramis E (2016). PD-1 blockade expands intratumoral memory T cells. Cancer Immunol Res.

[61] Piha-Paul Sarina Anne, Bennouna Jaafar, Albright Andrew, Nebozhyn Michael, McClanahan Terrill, Ayers Mark, Lunceford Jared K., Ott Patrick Alexander (2016). T-cell inflamed phenotype gene expression signatures to predict clinical benefit from pembrolizumab across multiple tumor types. Journal of Clinical Oncology.

[62] Topalian SL, Taube JM, Anders RA, Pardoll DM (2016). Mechanism-driven biomarkers to guide immune checkpoint blockade in cancer therapy. Nat Rev Cancer.

