# Supplementary material for: Anti-PD-1 monoclonal antibody MEDI0680 in a phase I study of patients with advanced solid malignancies
Source: J Immunother Cancer. 2019 Aug 22;7:225. doi: 10.1186/s40425-019-0665-2 (PMC6704567; doi:10.1186/s40425-019-0665-2)
Supplement: Supplementary file 1 — Figure S1. Study design and pharmacokinetic/pharmacodynamic assessment. (a) phase I study design. (b) Overview of pharmacokinetic and pharmacodynamic profile assessment. Figure S2. MEDI0680 binding and specificity for PD-1. (a) MEDI0680 binding to activated primary human T cells. (b) Binding specificity of MEDI0680 to recombinant human proteins that share amino acid sequence homology with PD-1. Figure S3. Inhibition of ligand binding to native PD-1 by MEDI0680. (a) Blockade by MEDI0680 of recombinant human PD-L1 or (b) recombinant human PD-L2 binding to CHO cells expressing human PD-1 protein. Figure S4. In vitro T-cell activation and cytotoxicity mediated by MEDI0680. (a) IFNγ release into cell culture media of allogeneic dendritic cell–T cell mixed lymphocyte reactions. (b) Cellular cytotoxicity mediated by EBV-reactive CD8 T cells over time, as determined by non-invasive electrical impedance measurement in an xCelligence RTCA MP instrument as a surrogate for cell death. Figure S5. Representative examples of flow cytometry of peripheral blood from patients treated with MEDI0680. (a) Ki67 staining in CD4+ and CD8+ T cells at cycle 1 day 1 pre-treatment (C1D1) and at cycle 1 day 8 post-treatment, as indicated. (b) HLA-DR and CD38 co-staining on CD4+ effector memory T cells (CD4+ TEM) at the same time points. Figure S6. Lack of correlation between changes in peripheral pharmacodynamic markers and objective clinical response. (a) Fold change in the indicated cytokine and chemokine markers in all cohorts or (b) only in the 10 and 20mg/kg cohorts or (c) the fold change in T-cell proliferation and CD4+ TEM CD38high HLA-DRhigh (activated) T cells with respect to objective clinical responses are shown. Table S1. Key eligibility criteria. Table S2. Patient characteristics and samples evaluated for pharmacodynamic analysis. Table S3. In silico identification of PD-1 paralogs using the protein Basic Local Alignment Search Tool BLASTp. Table S4. Study disposition (as-treated p [file 40425_2019_665_MOESM1_ESM.zip › Figure S1B.pdf]

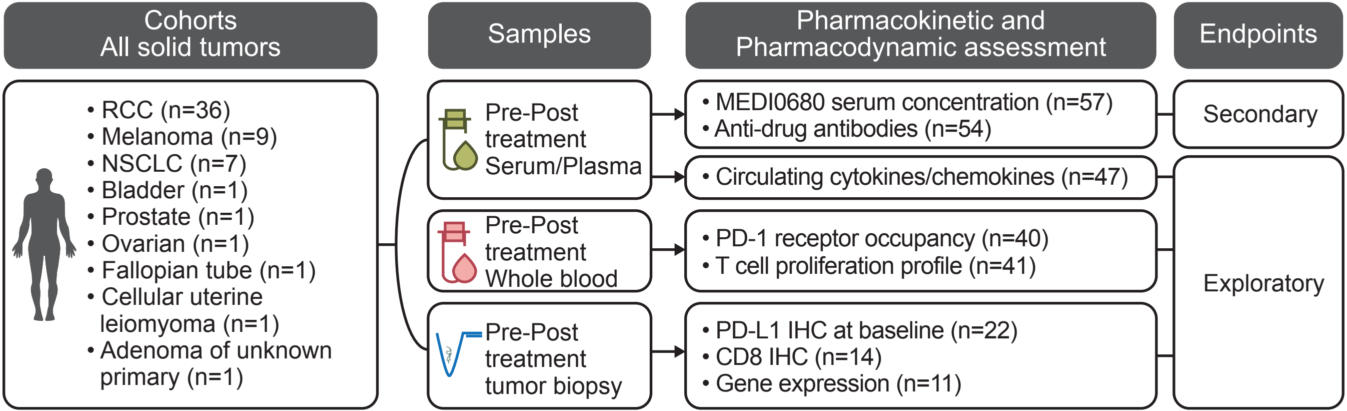
Cohorts
All solid tumors
Samples
Pharmacokinetic and
Pharmacodynamic assessment
Endpoints
• RCC (n=36)
• Melanoma (n=9)
• NSCLC (n=7)
• Bladder (n=1)
• Prostate (n=1)
• Ovarian (n=1)
• Fallopian tube (n=1)
• Cellular uterine leiomyoma (n=1)
• Adenoma of unknown primary (n=1)
Pre-Post treatment Serum/Plasma
Pre-Post treatment Whole blood
Pre-Post treatment tumor biopsy
• MEDI0680 serum concentration (n=57)
• Anti-drug antibodies (n=54)
• Circulating cytokines/chemokines (n=47)
• PD-1 receptor occupancy (n=40)
• T cell proliferation profile (n=41)
• PD-L1 IHC at baseline (n=22)
• CD8 IHC (n=14)
• Gene expression (n=11)
Secondary
Exploratory
